# Mapping and Visualization of Cancer Research in Indonesia: A Scientometric Analysis

**DOI:** 10.1177/10732748211053464

**Published:** 2021-10-26

**Authors:** Herindita Puspitaningtyas, Aufia Espressivo, Susanna H Hutajulu, Anis Fuad, Matthew J Allsop

**Affiliations:** 1 59166 Universitas Gadjah Mada, Yogyakarta, Indonesia; 2 Leeds Institute of Health Sciences, 4468 University of Leeds, Leeds, UK

**Keywords:** cancer research, cancer epidemiology, scientometric analysis, policy, Indonesia

## Abstract

**Introduction:**

The incidence of cancer and its prevalence are increasing in Indonesia. It is crucial to ensure national cancer policies are evidence-based and promote research. While cancer research is being conducted across Indonesia, the extent and focus of research activities are not known, with no existing synthesis of the cancer research landscape. We seek to address this gap by characterising trends in the extent and types of cancer research conducted in Indonesia.

**Methods:**

Scientometric study using descriptive analyses to determine annual growth patterns in publications across all cancer research literature from Indonesia. We developed a classification system for both research type and study design which was applied to all included publications. A visualisation software tool (VOSviewer) was used to explore the geographical distribution of research activity. The Wilcoxon rank-sum test was used to determine the influence of international collaboration on the impact factor of journals in which articles were published.

**Results:**

We retrieved 1773 cancer-related articles published by Indonesia-affiliated authors from 1961 to 2020, with notable year-on-year increases in the annual total number of published articles since 2015. Most articles (84.0%) were published by authors affiliated with institutions on Java Island. The most commonly published article type was basic research and discovery science (28.8%), using a one-group analytical study design (28.8%). International collaboration was significantly correlated with a higher h-index of the journal in which research was published (P < .0001, r = .317).

**Conclusion:**

An increase in the number and range of topics explored in cancer-related publications over time was identified. The summary of the current corpus of cancer-related research for Indonesia can be used to direct the development of the national cancer control plan alongside informing the national cancer research strategy. Our novel and feasible scientometric approach can be used to direct future national and regional mapping of cancer research.

## Introduction

Despite the development of cancer prevention and treatment methods, the morbidity and mortality of cancer are increasing rapidly worldwide.^1^ In 2020, breast, lung and colorectal cancer had the highest incidence globally, while lung cancer was the leading cause of cancer death.^2^ The increase in the burden of cancer, particularly in the context of low- and middle-income countries, is being driven by factors including population growth, ageing and lifestyle (e.g. diet and physical activity levels), as well as social and economic development. Broadly, a positive relationship exists between overall cancer incidence and the Human Development Index (HDI) measure,^3^ with a higher cancer incidence in both males and females in countries with higher HDI levels. However, in terms of cancer mortality, the risk is higher in countries ranked at lower levels of the HDI.^4^ Indonesia, a middle-income country with an approximate population of 270 million, has an increasing cancer burden. The latest data from Globocan for 2020 indicated a rise in new cancer cases to 141.1 per 100 000 population, with cancer deaths at 85.1 deaths per 100 000 population.^5^ Cancer is a leading cause of death due to non-communicable diseases, second only to cardiovascular diseases, accounting for 18.6% of the 686 532 premature deaths in 2016 due to non-communicable diseases in the country.^6^

To respond to the increasing cancer burden, the Minister of Health of the Republic of Indonesia re released a Ministerial Regulation which monitors and enforces a national prevention program for breast and cervical cancers; two highly-prevalent cancers in Indonesia.^7^ The program focusses on health promotion, namely outreach to the public or community groups in public places, and preventive programs, including mass screening, early detection and referrals across primary healthcare facilities. These programs are still highly centralized with their reach and quality yet to be evaluated with, for example, only 7.6% of primary health centres equipped to implement the programs.^8^ There are several provinces with a high number of cancer patients, such as South Kalimantan and North Sulawesi, which currently do not have health facilities with early detection capacity.^8^

In recent years, Indonesia has prioritised health systems strengthening through the establishment and implementation of comprehensive cancer service guidelines.^7^ This effort was followed by the enactment of the population-based cancer registry in 14 provinces, with 14% coverage of the whole population and enabling the generation of national cancer incidence data.^9^ This is in line with the current National Cancer Control Plan containing 13 strategic goals which include increasing the quantity of standardised health service facilities, trained human resources, quality of healthcare delivery, patient safety, and technology and resources across the provision of cancer care.^10^ Additional goals include increases in the extent of cancer research being conducted nationally, providing opportunities to enhance evidence-informed policy and scope to explore co-production, collective ownership and the value of localised health policy and systems research for cancer care.^11^ Each year, in excess of 40 000 studies relating to cancer are published in scientific journals globally,^12^ although the contribution of outputs from Indonesia is not clear. Despite government targets to increase cancer research capacity and outputs in Indonesia, the extent, types and study designs of published cancer research literature from Indonesia is not known.

In Indonesia, cancer services are developing and there is a need to ensure practice is underpinned by evidence.^13^ A comprehensive overview of cancer research in Indonesia can be used to determine the extent of alignment of publications with national policy and the most prevalent cancers, whilst enabling benchmarking of progress in cancer research with other low- and middle-income countries (LMICs). Through understanding existing research outputs, it will be possible to guide future research activity to address identified gaps and ensure its relevance to decision makers at multiple levels of influence (e.g. professional groups and organisations can use local systems to validate evidence, or findings can be tailored and framed to influence and inform clinical practice).^14,15^ Such activities can encourage evidence-based practice which is essential to underpin healthcare decision making.^13,14^ To address the lack of evidence on the extent and types of cancer research from Indonesia, we conducted a scientometric analysis of published literature. We sought to characterise existing research that has been conducted to date, determine trends in publications over time and identify any gaps to guide and inform future national research strategies.

## Methods

Our objectives were to i) determine the extent of cancer research publications in Indonesia, ii) identify the number of publications across research organisations in Indonesia, iii) characterise research types and study designs reported in cancer research publications in Indonesia, iv) explore relationships across research publications through keyword co-occurrence and v) explore the extent and impact of international collaboration underpinning cancer research publications in Indonesia.

### Bibliographic Search

We developed detailed search strategies with input from information specialists at the University of Leeds, UK, to identify studies reporting cancer research that had taken place in, or had a focus on, Indonesia. The search terms included cancer, neoplasm, associated cancer treatments, and Indonesia, around which a strategy comprising both medical subject headings (MeSH) and keywords was developed. Searches were conducted in MEDLINE (via PubMed), EMBASE (via Ovid) and Web of Science, in November 2020. An example search strategy can be found in the supplementary attachment, Supplementary Table S1. This sought to achieve coverage of medical research literature and increase coverage through searching multiple databases. The inclusion criteria included cancer research conducted by or with at least one author affiliated with an Indonesia-based organisation, using any study design, which had been conducted in any year (i.e. no limits were placed on year of publication). Studies were included that were published in the English language. We excluded conference abstracts. Data were extracted for all studies from the beginning of records indexed in the databases, to November 30, 2020. Data were extracted for multiple fields and exported into a comma-separated values format and accessed using Microsoft Excel. The data extraction form included headings for a unique assigned study ID (sequentially numbered from 1), PubMed ID number, publication year, title, abstract (for study classification purposes), author affiliation, journal title and ISSN. The first author affiliation was further reviewed to map the organisation, province and country. In the case where double affiliation consisting of Indonesian and international institutions was found, we opted to categorise the first author affiliation according to their Indonesian organisation. If the double affiliation consisted of both Indonesian affiliation (i.e. university and hospital), we included both organisations for classification purposes. The journal title information was merged with Scimago Journal Rank (SJR) data.

### Development of Classification of Research Type and Study Design

There were no identified frameworks to support the alignment of included publications against types of cancer research. A classification was developed comprising four groupings, formed through discussion with cancer research experts across research institutions of authors, and multiple iterations by the research team. The resulting research types used in this study align with a translational pathway from basic science through to the generation and eventual evaluation of clinically testable interventions. Table 2 outlines examples of the research foci across the different research types. Classification of study design was informed by the Centre for Evidence-Based Medicine (University of Oxford, UK) classification of study design, with further categories added to code narrative and review articles alongside experimental designs used for basic biology, genetics and immunology, and biomarker discovery.

### Classification and Coding of Included Articles

Using data contained in the data extraction form, two researchers (AE and HP) independently reviewed the title and abstract for each article and categorised each article according to one of four research types and one of eight study designs (see Table 1 for categories and examples). Where disagreements arose in coding, a third reviewer (MJA) was involved and a consensus was achieved through discussion. During the classification of studies, those articles not describing research on cancer in Indonesia were removed. The a priori defined categories for the research type and study design were used to code each included article (Table 1).

**Table 1.** Overview of categories and definitions used for coding research type and study design for included studies.

Research Type	Examples of Categories of Research Coded under Each Research Type
1 - Basic research & discovery science	• Studies focussing on DNA, cells, proteins, molecules, signalling pathways, tumour biology, tumour immunology and stem cells • Early exploration that may have future therapeutic potential or diagnostic tools • Computational modelling work • Study trials in mice/animals
2 - Clinical observations & experimental early-phase studies	• Exploring whether new treatments, medications and diagnostic techniques are safe and effective in patients • Clinical trials phase 0 – 2. Early-phase trials typically aiming to find out, for example, the correct dosage, frequency, side effects and toxicities, risk, and pharmacokinetics
3 - Clinical evaluations & trials	• Comparisons between the most effective known treatment for a specific type or stage of cancer with a new approach (e.g. new drug, or combination of drugs, or a different way of using established therapies) • Clinical trial phases 3 and 4 • Case reports
4 - Health services, policy, public health and implementation research	• Methods to promote the adoption and integration of evidence-based practices, interventions, and policies into routine health care and public health settings to improve the impact on population health, including epidemiological studies • Exploring service experience (patients and caregivers) and care being delivered (health professionals, policymakers)
**Study Design**	
1 Review and narrative articles	Review article (systematic review, meta-analysis or literature review); Editorial paper, letter to the editor, short communication, guideline(s)
2 Basic biology, genetic and biomarker investigation	Basic biology; genetics and immunology; biomarker discovery (non-drug related)
3 Preclinical testing	Preclinical studies (in vitro, in vivo and in silico profiling using computer models of the drug-target interactions); animal studies and models
4 Descriptive and within-group	Descriptive and within-group study design (case report, case series, cross-sectional study, longitudinal study, modelling studies with one group)
5 One-group analytic	One-group analytical study, including prevalence from clinical and primary data
6 Analytical	Analytic study (ecological study, cross-sectional two-group study with comparison between groups, cohort study, economic analytic modelling)
7 Intervention	Intervention study (trials in humans including parallel-group and crossover designs)
8 Qualitative	Qualitative study

### Metrics for Assessing Ranking of Journals Used for Publication of Cancer Research

The SJR was used to classify all journals in which included studies had been published. The SJR was designed to be a measure of the scientific influence of scholarly journals, accounting for both the number of citations received by a journal and the importance or prestige of the journals from which citations occur. All journals included in the SJR database are allocated to quartiles, with quartile 1 (or Q1) accounting for the top 25% of journals for SJR. All included articles were assigned their respective quartile as registered in the Scimago database where this was available for 2019. Where a rank was not available for 2019, the most recently available quartile allocation was used from 2017 or 2018 or otherwise a rank was not applied.

## Data Analysis

### Descriptive Analysis

To address objectives i, ii and iii, descriptive analysis was undertaken to determine the publication trends by determining the frequency of article publications by year, alongside exploring trends in the research type and study design by year. Frequencies were calculated for the journals in which included articles were published alongside institutions to which first authors were affiliated. Descriptive analysis was performed using Microsoft Excel and Tableau Desktop 2020.2.

### Visualisation Analysis

To address objective iv, we explored relationships between included articles through the generation of bibliometric network visualisations. In the data preparation, the bibliographic information of all included articles was extracted to Mendeley Desktop 1.19.4. Articles belonging to each category were grouped manually to generate a .ris file across 4 research types. To explore keywords used to index studies included in the scientometric analysis, all the keyword terms were extracted from included studies and were filtered to only include those with a minimum of 10 occurrences. From included studies, keywords were extracted through a VOSviewer built-in text mining function using .ris data. A thesaurus file was created to group similar keywords. VOSviewer used the keywords of included articles to generate co-occurrence tables and clustered based on the co-occurrences. Two words are defined as co-occurring if they appear in the same document. In the generated figures, the size and colour of circles represent their frequency of occurrence and clustering of individual keywords. The distance between circles reflects how frequently they co-occur (i.e. the closer words are together suggests they occur more frequently together). Colours of clusters are used to represent keywords with strong co-occurrence links. Co-occurrence values were calculated for the most frequently occurring 50 keywords (excluding the check tags and additional keywords in Appendix A) that appeared across included articles. Five figures were produced, referred to as Social Network Analysis (SNA) maps. The first included all articles included in the scientometric analysis to provide an overview of all cancer research in Indonesia. Four additional figures were produced, exploring the co-occurrence of keywords across studies coded across each of the four research design types. All visualisation networks were limited to studies published in the last 10 years to reflect the most recent literature.

### Statistical Analysis

To address objective v, and determine the impact of international collaborations on the SJR of publications, we used RStudio version 1.3.1056 to conduct a Wilcoxon rank-sum test (Mann Whitney U test) to determine whether there were differences in the rank of journals where international collaborators were the first author on publications.

### Ethics Statement

Ethical review and approval were not required for this study because it uses publicly available data from scholarly documents.

## Results

**Figure 1.**
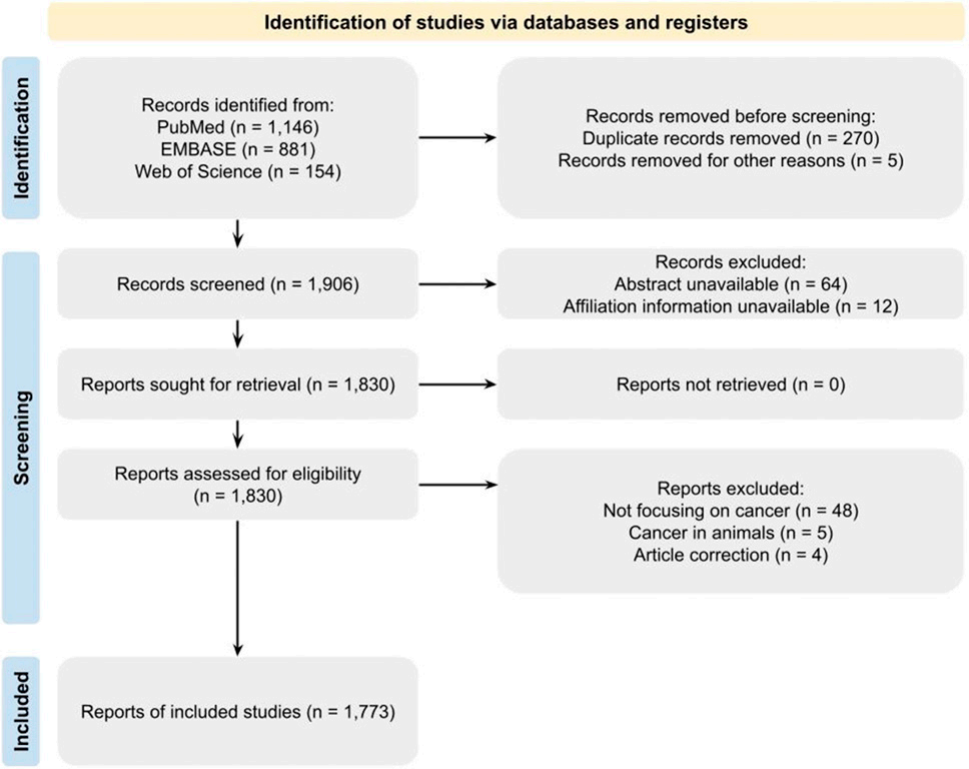
Preferred reporting items for systematic reviews and meta-analysis flow diagram (PRISMA flow diagram).

The search of the literature returned 2181 articles. After screening articles, 1773 papers were included (Figure 1).

### Extent of Publications and Source of Research

**Figure 2.**
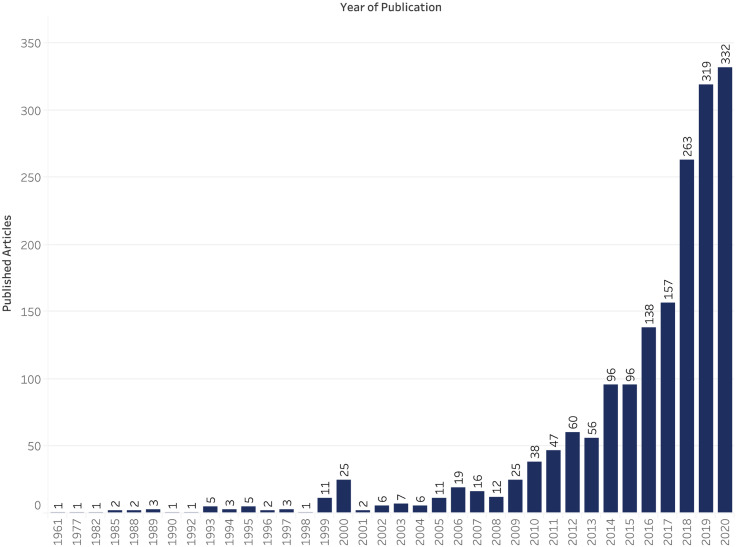
Cancer research publication per year.

**Figure 3.**
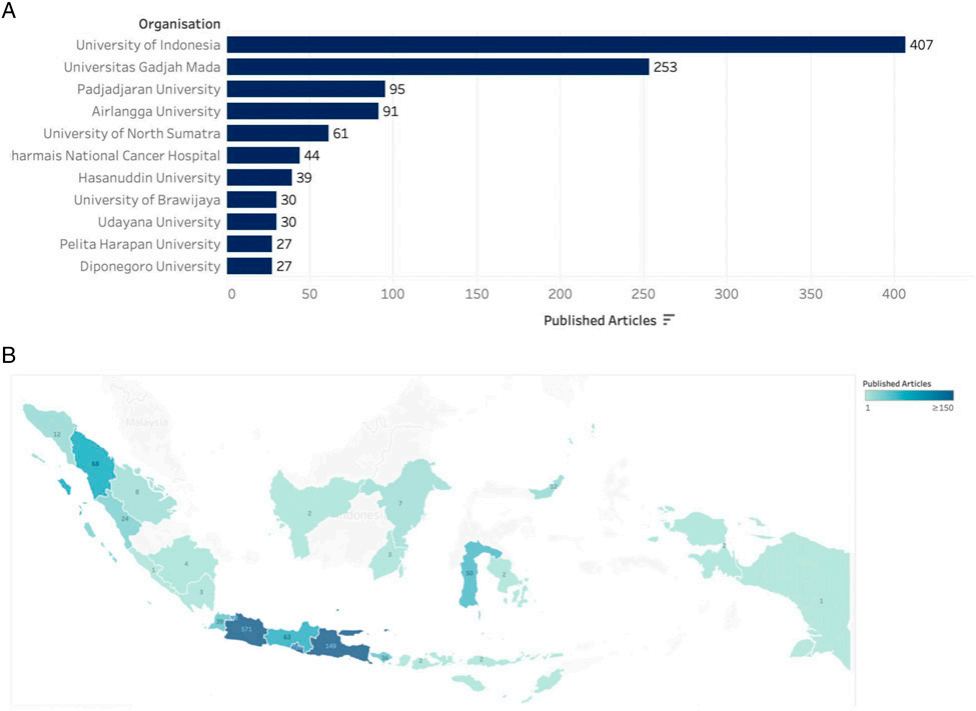
(a) Frequency of publication accross research organisations (Indonesia); (b) geographical depiction of location of lead authors across Indonesia.

Since the year 1999, there has been an increasing number of cancer research articles published in Indonesia (Figure 2), with a year-on-year increase since 2015 and a total of 332 articles published in 2020. The majority of articles (n = 1493) involved first author affiliations with a research organisation in Indonesia, with the main contributors including the University of Indonesia (n = 407, 27.3% ), Universitas Gadjah Mada (n = 253; 14.3%) and Padjadjaran University (n = 95; 5.4%) (Figure 3(a)). The three institutions account for 56.7% of all published cancer research from research organisations in Indonesia. This in part explains the greater frequency of articles depicted as arising from the island of Java, where the three institutions are based (Figure 3(b)), as opposed to the 16.0% published by institutions from outside Java island.

### Research Type and Study Design

Table 2 outlines the types of study design reported across included publications, aligned to each of the four research types. The most common research type was basic research and discovery science (n = 512; 28.9%) and the most common study designs were one-group analytical studies (n = 511; 28.9%) and descriptive and within-group designs (n = 359; 20.2%).

**Table 2.** Distribution of articles based on the research type and study design.

*Research type*	*Study Design*								
	Review/Narrative	Basic Biology/Genetics	Preclinical	Descriptive/Within-Group	One-Group Analytic	Analytical	Intervention	Qualitative	Total
1 - Basic research & discovery science	56	222	169	5	38	22	-	-	**512**
2 - Clinical observations & experimental early-phase studies	32	3	6	153	191	80	4	-	**469**
3 - Clinical evaluations & trials	29	-	-	76	109	72	35	-	**321**
4 - Health services, policy, public health and implementation research	87	-	-	125	173	28	5	53	**471**
**Total**	**204**	**225**	**175**	**359**	**511**	**202**	**44**	**53**	**1773**

**Figure 4.**
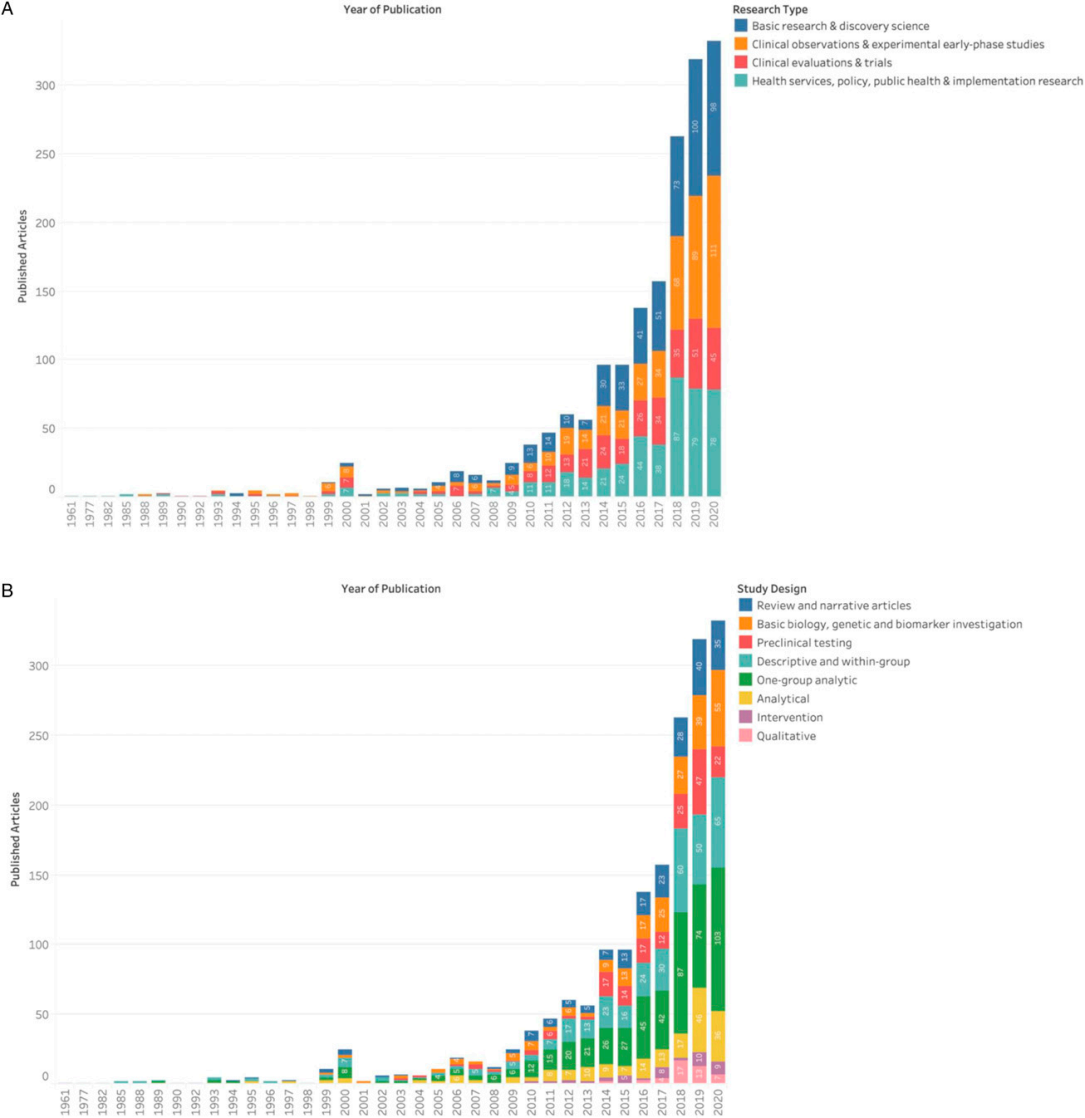
(a) Publication frequency across research stages; (b) Publication frequency across study designs.

Figure 4(a) provides an overview of the trends in publications aligned with four research types. All research types have seen progressive growth in the number of publications over time, with marked increases for all research types in the last 10 years. Basic research and discovery science is the most common type reported in publications since 2014. Research types of both clinical observation and health services research have seen 4-fold and 3-fold increases in publications, respectively, between 2017 and 2020. Clinical evaluation research has seen a steadier trend over time, accounting for 13.6% of all published articles in 2020. Similarly, general growth across all categories of study design is displayed in Figure 4(b).

### Keyword Co-occurrence and Relationships Across Publications

**Figure 5.**
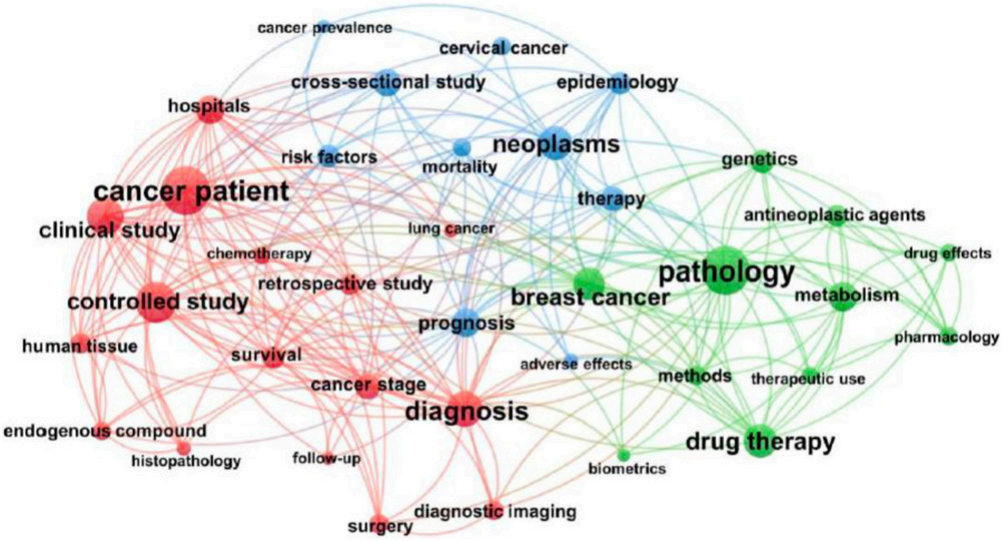
Social network analysis map of Indonesian cancer research.

Figure 5 shows an SNA map that includes all studies between 2011 and 2020, comprising 1564 articles (88.2% of all publications), were characterised into three clusters: Cluster 1 (red) outlines co-occurrence across controlled and clinical studies focussing on cancer patients, with only lung cancer included as a focus on cancer type. Cluster 2 (blue) outlines multiple studies with a focus on cervical cancer and neoplasms broadly and epidemiology and prognosis, with cross-sectional studies commonly reported. Cluster 3 (green) outlines research focussed on pathology and drug therapies, with a focus on pharmacology, alongside a specific focus on breast cancer.

**Figure 6.**
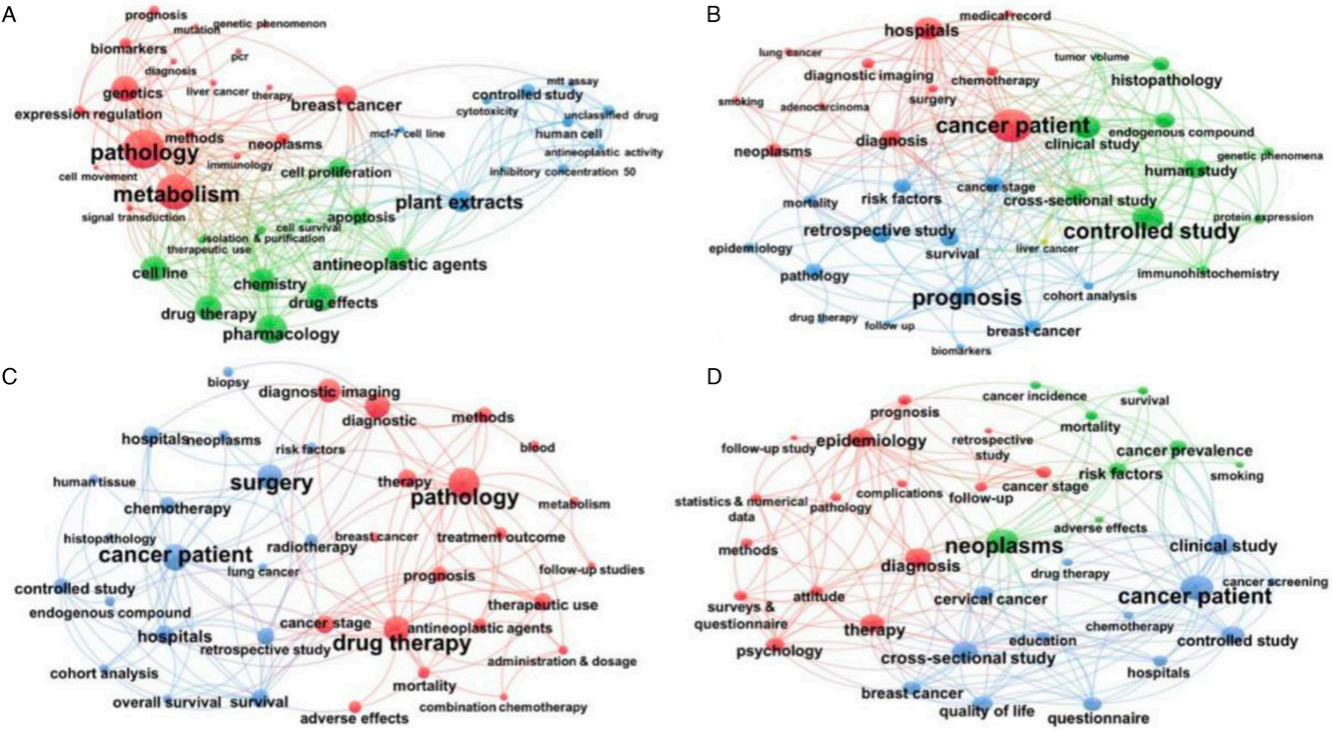
Social network analysis maps of cancer research articles for: (a) Basic science/discovery research; (b) Clinical observation/experimental early-phase studies; (c) Clinical evaluations and trials; (d) Health services, policy, public health and implementation research.

Additional SNA maps were generated for each of the categorised research types. For basic research and discovery science, clusters were focussed around pathology and genetics research, pharmacology and drug effects, and controlled and in-vitro studies (Figure 6(a)). Clinical observation and experimental early-phase studies (Figure 6(b)) were clustered around controlled and major clinical studies adopting a range of study design approaches, and include diagnostic and survival studies. Studies focussing on clinical evaluations and trials (Figure 6(c)) reflect multiple clusters, with the most common including pathology (with a focus on prognosis and mortality), drug therapies and diagnosis (including survival analyses and clinical studies exploring a range of treatment modalities and their side effects). Studies exploring health services, policy, public health and implementation research (Figure 6(d)) outline three clusters, including diagnostic and major clinical studies, epidemiology (including cancer incidence and prevalence) and psycho-social research (including a focus on quality of life associated with therapies).

### Quality of Publications and Extent and Impact of International Research Collaborations

**Figure 7.**
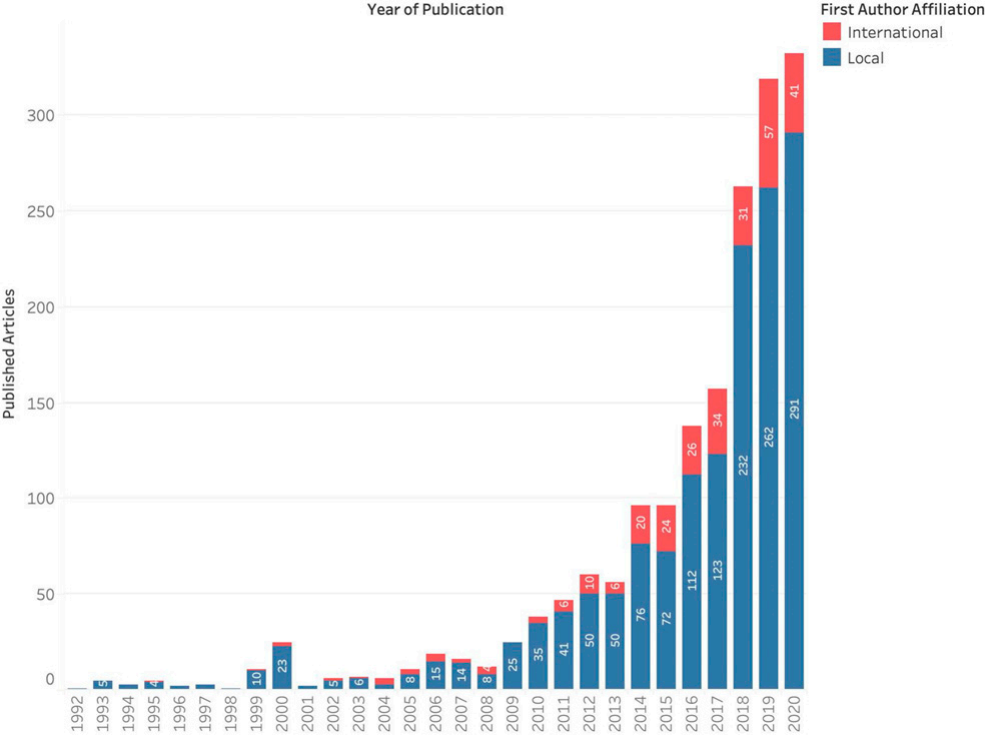
Proportion of publications including international collaboration.

Figure 7 highlights the increasing number of publications that include international collaborations. Over one tenth (41 of 332 articles; 12.3%) of all publications in 2020 were first-authored by an international collaborator. Across all included studies, first author affiliations outside Indonesia (n = 280) are reported across 33 countries but are predominated by researchers from Japan (n = 85; 30.4%), the Netherlands (n = 44; 15.7%), the United States of America (n = 21; 7.5%), Taiwan (n = 14; 5.0%) and Malaysia (n = 12; 4.3%).

**Figure 8.**
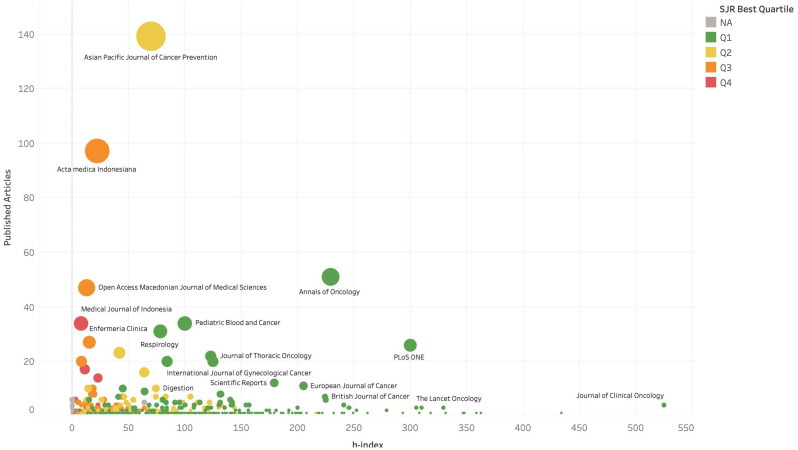
Frequency of publications in journals against the average h-index of journals.

We identified that articles on cancer research in Indonesia were being published in a range of journals with differing SJR rankings (Supplementary Figure S1). Publications were reported across the first (n = 664; 37.5%), second (n = 461; 26.0%), third (n = 445; 25.1%) and fourth (n = 131; 7.4%) quartiles, with over 63.5% of all articles published in the highest two quartiles. Seventy-two (4.0%) publications were published in journals that are either unlisted in SJR or listed but not assigned a quartile ranking. When exploring the number of articles against the h-index of journals in which articles are published, Figure 8 reflects that the majority of articles are published in journals with lower impact factors.

**Figure 9.**
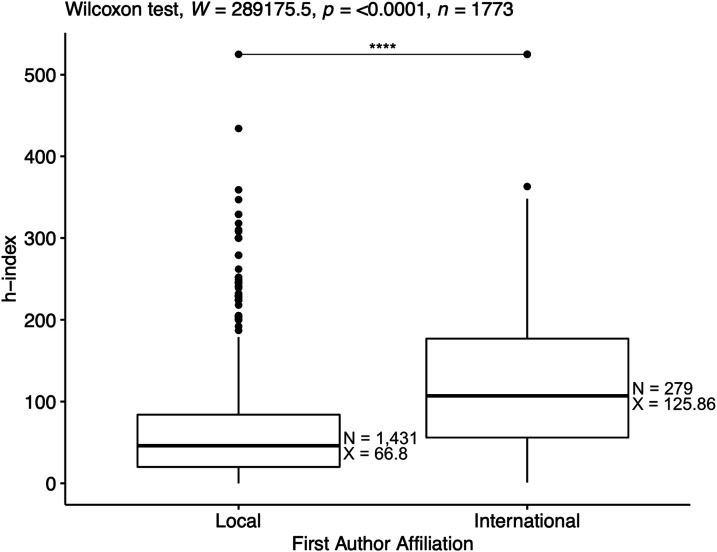
Box plot of the average H-index for journals for article publications.

To determine the impact of international collaborations on the overall quality of publication outputs, a skewness and kurtosis calculation was performed to determine the normality of data, which resulted in 1.85 and 7.56, respectively, indicating the data was not normally distributed. A Wilcoxon rank-sum test (Figure 9) determined that publications developed in partnership with international collaborators had a statistically significant higher h-index for the journal in which outputs were published (P < .0001, effect size r = .317). The median h-index was 100 for publications in partnership with international collaborators compared to 44 for those with local or national collaborators (Supplementary Table S2).

## Discussion

This study characterises the corpus of cancer research for Indonesia to date, identifying year-on-year increases in publications since 2009 and a marked increase in the annual number of publications over the last 5 years. Publications include research types across the cancer research pathway, with the commonest being basic research and discovery science. There has been an array of study designs used across publications, with descriptive, within-group designs and one-group analytical studies being the most frequently reported. There is a predominance of research publications from institutions located on Java, an island that is the current geographic and economic centre of Indonesia. It is also the location of the three leading institutions for cancer research output nationally. Our study also identified the increasing involvement of international research partners in cancer research, with this having a significant effect on the impact factor of journals in which articles are published. Our findings contribute to a lack of evidence for Indonesia, and more broadly LMICs, regarding the extent and diversity of publications relating to cancer research. There has been a limited number of bibliometric analyses of national cancer research activities published, largely focussed on specific cancer types from Portugal and Peru,^16,17^ with country-level analyses identified for Iraq and Iran,^18,19^ and those mapping research across wider regions including Central and Eastern Europe, the Russian Federation and Central Asia.^20^ Aligned with previous research,^20^ our study indicated that research outputs reflected the national cancer burden for Indonesia. Social network analysis highlighted that the three most common cancers in Indonesia, breast, cervical and lung,^6^ were the most common focus across research outputs.

Currently, the largest number of cancer research publications worldwide is generated by the US, China and Japan,^21^ with the scarcity of information published from Indonesia previously highlighted.^22^ Although the number of publications produced by Indonesian researchers was still comparably lower than the aforementioned countries, a steady increase can be observed since 2010. The findings from this study can enable benchmarking of the quantity and quality (using journal impact factor as a proxy measure) of cancer research publications for Indonesia. It also provides a methodology for further monitoring political declarations for increased cancer research nationally. Existing cancer research publications are diverse and reflect multiple study designs across all research types across the cancer research pathway. Starting in 2014, there has been a large increase in cancer research with a focus on the research type, health services, policy, public health and implementation research. This is now the second most common category of cancer research in Indonesia. Emerging evidence is making clear the impact and burden of cancer on patients and their families, with half of patients experiencing financial catastrophe and a third dying within one year of a cancer diagnosis.^23^ Increasing levels of research focussed on service delivery may provide evidence to guide and optimise existing delivery of care through, for example, further exploration of the needs and preferences of patients with cancer and their caregivers. Furthermore, the development of implementation science research can support efforts to translate research into accessible and actionable forms of evidence for policymakers and practitioners.^24^ To sustain and extend outputs with direct relevance to public health and policy, linkages with universities, ministries and international agencies such as the World Health Organization will be critical for building sustainable high-quality programmes. Such approaches have been successful in, for example, enhancing capacity for epidemiology training^25^ and medical education^26^ in Indonesia, which themselves may have contributed to the increase in public health and policy research on cancer noted in this study for Indonesia since 2014.

Findings from this study suggest that the h-index of the journal in which articles are published is higher where international collaborators lead as first authors. There may be multiple factors underpinning this trend, for example, differences in the conduct or reporting of research, alongside the potential availability of funding to cover article processing charges providing a wider option of journals for submission of manuscripts. In future scientometric analyses, assessing changes in the representation of local authors where international collaborations occur could be a useful metric for understanding power dynamics within global health research collaborations in Indonesia.^27^ Alongside efforts to ensure equity in collaborations, our analysis highlighted a predominance of research outputs from three institutions all located on the island of Java. This may present an opportunity to explore the scope for within-country capacity strengthening and mentoring initiatives of institutions by nationally leading cancer research organisations to ensure increased cancer research activity, including data collection and participant representation, from across the country. For Indonesia, there may also be scope to increase South-South partnerships in cancer research, providing opportunities for partner countries to draw on and provide technical resources for researchers while maintaining leadership over the resulting work, aligned with wider efforts to improve responses to non-communicable diseases which includes cancer.^28^

Our work presents a template for characterising and defining cancer research conducted to date at a country level, enabling comparison and benchmarking, alongside gauging the extent of research activity when using publications as a proxy measure. Our methods and analysis utilised freely available software, ensuring increased accessibility and feasibility for replication for different country settings. Adoption of the study methodology by different countries presents opportunities for comparative benchmarking, alongside exploring alignment between cancer outputs and national strategy and policy development. Furthermore, we conducted a comprehensive search of research databases and aligned our methodology with guidance on optimal database combinations for literature searching in systematic reviews.^29^ Our study has limitations. Due to resource constraints, we could not extend database searches to include grey literature or articles published in local languages due to the increasing number of local journals indexed in the Directory of Open Access Journals.^30^ Whilst we are confident that a large proportion of the cancer research literature for Indonesia is presented, it may not comprise all publications in their entirety.

## Conclusions

This is the first national overview of cancer research in Indonesia, performed using novel classifications of research design and study type and enabling alignment of research activities with national cancer strategy. We outlined an increase in publications for the country, particularly from institutions located in Java, with a notable year-on-year increase in publications nationally since 2014. Basic research and discovery science remains the most common research type being explored in Indonesia, although we observed an increase in studies on health services, policy, public health and implementation research. Analysis of international collaborations highlighted opportunities for within-country research capacity strengthening to support research organisations outside Java. Our study methodology presents a feasible and easily adapted approach to map cancer research trends on a national level. The approach and the insights it affords could serve as the basis to direct further cancer-related research and development nationally, especially in an LMIC with limited resources such as Indonesia.

## Supplemental Material

sj-pdf-1-ccx-10.1177_10732748211053464 – Supplemental Material for Mapping and Visualization of Cancer Research in Indonesia: A Scientometric Analysis Click here for additional data file. Supplemental Material, sj-pdf-1-ccx-10.1177_10732748211053464 for Mapping and Visualization of Cancer Research in Indonesia: A Scientometric Analysis by Herindita Puspitaningtyas, Aufia Espressivo, Susanna H Hutajulu, Anis Fuad and Matthew J Allsop in Cancer Control
