# Supplementary material for: Mapping and Visualization of Cancer Research in Indonesia: A Scientometric Analysis
Source: Cancer Control. 2021 Oct 26;28:10732748211053464. doi: 10.1177/10732748211053464 (PMC8554557; doi:10.1177/10732748211053464)
Supplement: sj-pdf-1-ccx-10.1177_10732748211053464 – Supplemental Material for Mapping and Visualization of Cancer Research in Indonesia: A Scientometric Analysis [file sj-pdf-1-ccx-10.1177_10732748211053464.pdf]

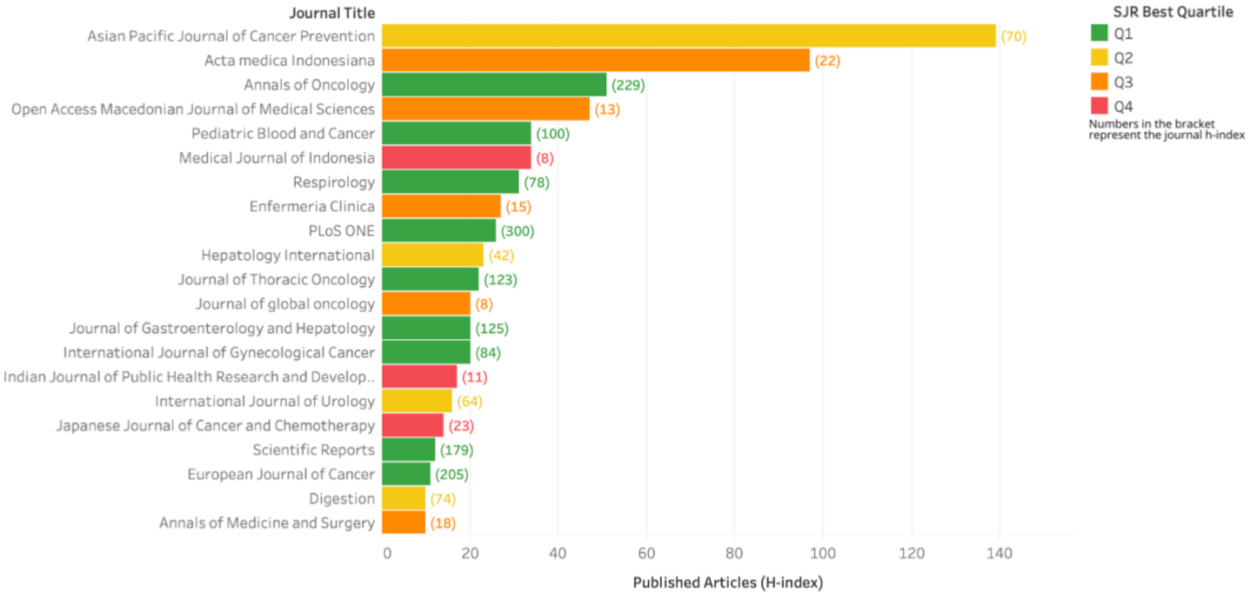

Figure S1. Classification of the top 20 most common journals for cancer research in Indonesia using the SJR rankings

Table S1. Example search strategy used to identify relevant articles for Embase

1 exp Neoplasms/ or exp Breast Neoplasms/ or exp Oncology/ or exp Chemotherapy/ (5093335)

2 (neoplasms or oncology or palliative care).mp. [mp=title, abstract, heading word, drug trade name, original title, device manufacturer, drug manufacturer, device trade name, keyword, floating subheading word, candidate term word] (619539)

3 exp Radiation Therapy/ (604418)

4 chemotherapy.mp. or exp Chemotherapy/ (913051)

5 (radiotherap* or radiation or radiochemotherap* or chemoradi* or chemotherap*).mp. [mp=title, abstract, heading word, drug trade name, original title, device manufacturer, drug manufacturer, device trade name, keyword, floating subheading word, candidate term word] (1951201)

6 (neoplasm* or cancer* or carcinoma* or tumour* or adenocarcinoma* or leukemi* or leukaemi* or lymphoma* or tumor* or malignan* or myeloma*).mp. [mp=title, abstract, heading word, drug trade name, original title, device manufacturer, drug manufacturer, device trade name, keyword, floating subheading word, candidate term word] (6106335)

7 1 or 2 or 3 or 4 or 5 or 6 (7193349)

8 indonesia.ti,ab. (18873)

9 7 and 8 (1884)

First Author Affiliation	N	Mean	Median	IQR	SD
International	279	125.857	107	121	92.432
Local	1,431	66.828	46	64	65.583
Total	1,710	76.46	59.5	78	73.92

Table S2. Summary statistics of the journal h-index of published articles based on the first author affiliation.
